# Bovine colostrum prevents formula-induced gut microbiota dysbiosis in preterm pigs

**DOI:** 10.1038/s41390-024-03379-x

**Published:** 2024-07-08

**Authors:** Lin Yang, Yan Hui, Thomas Thymann, Dennis Sandris Nielsen, Ping-Ping Jiang, Per Torp Sangild

**Affiliations:** 1 Section for Comparative Pediatrics and Nutrition, Department of Veterinary and Animal Sciences, University of Copenhagen, Frederiksberg, Denmark; 2 Department of Food Science, University of Copenhagen, Frederiksberg, Denmark; 3 Department of Neonatology, Rigshospitalet, Copenhagen University Hospital, Copenhagen, Denmark; 4 Department of Pediatrics, Odense University Hospital, Odense, Denmark; 5 Faculty of Theology, University of Copenhagen, Copenhagen, Denmark

## Abstract

**Background:**

Preterm birth and formula feeding increase the risk of necrotizing enterocolitis (NEC), a gut inflammatory disease known to be associated with gut microbiota (GM) changes in infants. Supplemental bovine colostrum may protect against formula-induced NEC via GM changes. We hypothesised that feeding colostrum before, after, or during formula feeding affects NEC sensitivity via changes to GM.

**Methods:**

Colonic GM (profiled by 16S ribosomal RNA gene amplicon sequencing) was compared in preterm pigs fed colostrum for 4 days, either before, after, or together with formula feeding for 4 days. Correlations between GM and gut parameters were assessed on day 5 or 9.

**Results:**

Both exclusive and partial colostrum feeding induced higher GM diversity, lower *Enterococcus* abundance, and improved intestinal maturation parameters (villus structure, digestive enzyme activities, permeability), relative to exclusive formula feeding (all *p* < 0.05). Across feeding regimens, *Enterococcus* abundance was inversely correlated with intestinal maturation parameters. Conversely, there was no correlation between GM changes and early NEC lesions.

**Conclusion:**

Bovine colostrum inhibits formula-induced *Enterococcus* overgrowth and gut dysfunctions just after preterm birth but these effects are not causally linked. Optimising diet-related host responses, not GM, may be critical to prevent NEC in preterm newborn pigs and infants.

**Impact:**

- Supplement of bovine colostrum to formula feeding modified the gut microbiota by increasing species diversity and reducing *Enterococcus* abundance, while concurrently improving intestinal functions in preterm pigs.
- Diet-related changes to the gut microbiota were not clearly associated with development of necrotizing enterocolitis (NEC) in preterm pigs, suggesting that diet-related gut microbiota effects are not critical for diet-related NEC protection.
- The study highlights the potential to use bovine colostrum as a supplement to formula feeding for preterm infants lacking human milk.

## Introduction

Early postnatal feeding with an optimal milk diet is important for the health and development of preterm infants.^1^ Mother’s own milk (MOM) is the preferred diet,^2^ but not always available or sufficient in the first days of life. Donor human milk (DHM) is widely used in some countries but unavailable in others. The alternative, infant formula, is widely used but is associated with increased risk of gut complications (feeding intolerance) and necrotizing enterocolitis (NEC), a serious gut inflammatory disease common in very preterm infants (<32 weeks gestation).^3,4^

Bovine colostrum, the initial milk of dairy cows, is rich in protein and various bioactive factors that are also found in MOM, such as lactoferrin (LF), immunoglobulins (Igs), insulin-like growth factors (IGFs) and lysozyme.^5^ Numerous pre-clinical studies, using preterm pigs as model for infants, demonstrated that bovine colostrum has potential as an alternative to MOM or DHM as the first nutrition for preterm infants.^6–11^ Feeding bovine colostrum just after birth, in various amounts, with or without parenteral nutrition, and with or without added systemic immunity, protects against NEC-like lesions and improves gut maturation by increasing intestinal villus growth, mucus production, absorptive capacity, brush border enzyme activities and reduce gut permeability and tissue proinflammatory cytokines.^6–11^ In pilot preterm infant studies, bovine colostrum was well tolerated, increased enteral protein intake and appeared safe relative to human milk or formula.^12,13^ A larger trial of very preterm infants (*n* = 350) indicated that bovine colostrum supplemented to MOM or formula just after birth, reduced feeding intolerance, with no effects on body growth, time to full feeding (120 mL/kg/d) or clinical variables.^14^ Here, supplemental colostrum was fed before/after, or alternating with MOM or formula, rather than exclusively (to maximise MOM intake and avoid protein overload)^14^ and gut microbiota (GM) effects were limited (unpublished observations). However, a sub-group of infants fed larger amounts of bovine colostrum with no MOM (up to 50 mL/kg/d in the first 1–2 weeks) showed reduced time to full enteral feeding.^14^ It is unknown if bovine colostrum retains its gut-protective effects when combined with MOM or formula in various proportions. Further, it is unknown if such protective effects are closely related to diet-induced changes to the GM and/or to host effects on intestinal maturation.

The GM is associated with diet, gut health and NEC in preterm infants but causal relationships are not clear.^15^ Shortly after birth, colonising bacteria may interact with diet and host cells to promote the proliferation of intestinal epithelial cells and differentially affect immune responses.^16^ Therefore, NEC-related GM changes can be a predisposing factor or a consequence of NEC progression, or both.^3,17,18^ In contrast to term infants, preterm infants typically exhibit a GM characterised by lower species diversity, less *Bifidobacterium* and a predominance of *Enterobacteriaceae* (including *Klebsiella* and *Escherichia*), *Staphylococcus*, and *Enterococcus*.^16,19^ The overgrowth of these opportunistic pathogens has been suggested to lead to NEC or late-onset sepsis (LOS).^20^ Formula-fed infants have less *Bifidobacterium*^21^ and more *Enterococcus*,^22^ compared with preterm infants fed exclusive MOM. In preterm NEC-sensitive pigs, exclusive formula feeding affects GM composition and increases NEC sensitivity, compared with bovine colostrum.^6,8^ However, it remains uncertain if colostrum feeding before or after formula feeding, or as part of daily meals, affects the GM and its relationship to NEC and gut maturation. To address this question, two studies on pigs were previously conducted to assess the impact of colostrum feeding before or after formula feeding (each 4 days), or in combination with daily formula meals for 4 days.^23,24^ Both exclusive and partial colostrum feeding (before/after formula feeding or as >50% of daily meals) improved intestinal functions and NEC resistance, relative to pigs fed exclusive formula.^23,24^ Such NEC-like lesions in preterm pigs may reflect the early phase of NEC progression in preterm infants. Here we conducted a secondary analysis, partly using the same animals, to investigate the impact of mixed feedings with bovine colostrum on the colonic GM and relate GM effects to relevant host effects.^23,24^ We hypothesised that colostrum supplementation changes the GM in formula-fed preterm pigs and that such effects are closely associated with gut protection.

## Methods

### Animal procedure and sample collection

**Fig. 1:**
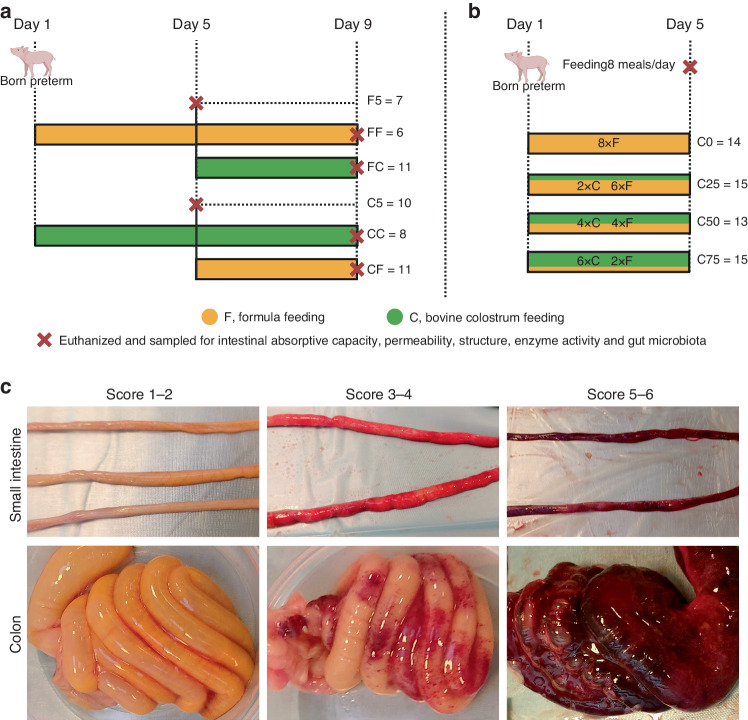
Study design and autopsy images with different necrotizing enterocolitis (NEC) score lesions. **a** Milk Shift (MS) study: Preterm pigs (*n* = 53) were fed bovine colostrum (C, green) or formula (F, yellow) until day 5, then euthanized (red cross) or fed with C or F until day 9 before euthanasia. This resulted in six groups: C or F until day 5 (C5 = 10, F5 = 7), C or F until day 9 (CC = 8, FF = 6), F before C (FC = 11) and C before F (CF = 11). **b** Mixed Milk (MM) study: Preterm pigs (*n* = 57) were fed increasing proportion of the eight meals per day as C, relative to F, either no C (C0 = 14), 25% (C25 = 15), 50% (C50 = 13) or 75% C (C75 = 15). **c** Representative autopsy images of small intestine and colon with different NEC score lesions: 1, normal without any lesion; 2, local hyperaemia, inflammation and oedema; 3, hyperaemia, extensive oedema and local haemorrhage; 4, extensive haemorrhage; 5, haemorrhage, local necrosis and pneumatosis intestinalis; 6, extensive necrosis, haemorrhage and pneumatosis intestinalis.^25^

Two experiments were conducted on preterm pigs and described in detail previously.^23,24^ In both experiments, preterm pigs were delivered by caesarean section at 90% of gestation from Danish Landrace × Large White × Duroc sows. In the Milk Shift study (MS study, Fig. 1a), preterm pigs (*n* = 53 available for GM analyses) from four sows were allocated into two groups fed bovine colostrum (C) with ColoDan powder (Biofiber-Damino, Gesten, Denmark) or formula (F) with ingredients including protein (DI-9224 whey protein isolate and Miprodan 40, Arla Foods Ingredients, Århus, Denmark), carbohydrate (Fantomalt, Nutricia, Amsterdam, the Netherlands), lipids (Liquigen-SHS and Calogen, Nutricia, Amsterdam, the Netherlands), and vitamins and minerals (Seravit-SHS or Phlexy-Vits, Nutricia, Amsterdam, Netherlands) until day 5 of life. Supplementary Table 1 shows the composition of the C and F feeding used in the experiments. On day 5, pigs were either euthanized or continued with the same feeding or shifted to the alternative diet (C or F) until day 9. In all, this resulted in six groups: Colostrum feeding until day 5 (C5, *n* = 10), formula feeding until day 5 (F5, *n* = 7), colostrum feeding until day 9 (CC, *n* = 8), formula feeding until day 9 (FF, n = 6), colostrum feeding to day 5 + formula feeding to day 9 (CF, *n* = 11) and formula feeding to day 5 + colostrum feeding to day 9 (FC, *n* = 11). In the Mixed Milk study (MM study, Fig. 1b), caesarean-delivered preterm pigs (*n* = 57) from three sows were randomly allocated into four different feeding procedures with eight meals a day: Formula feeding only (C0, 0% colostrum, *n* = 14), two feedings with colostrum (C25, 25% colostrum, *n* = 15), four colostrum feedings (C50, 50% colostrum, *n* = 13) or six colostrum feedings (C75, 75% colostrum, *n* = 15). In both studies, pigs were fitted with orogastric feeding tubes and umbilical catheters within 3 h of birth and reared individually in incubators with protocols for adjustment of temperature, moisture and oxygen, as described previously.^23–25^ The details of parental and enteral nutrition of both studies were as previously described^23,24^ and macronutrient and energy levels were similar between C and F diets, as described in Supplementary Table 1. The feeding regimens and volume progression rates were designed to induce moderate NEC sensitivity within the first week of life, as per our previous extensive experience, using the preterm pig model in NEC studies.^6,8,10,23,24^ All animal procedures were approved by the Danish National Committee on Animal Experimentation (license no.: 2020-15-0201-00520), which is in accordance with the guidelines from Directive 2010/63/EU of the European Parliament.

At euthanasia, a macroscopic NEC score was given to three regions of the small intestine (proximal, middle and distal; 16, 50 and 84% length along the intestine, respectively) and colon according to a scoring system described previously (1 = absence of lesion; 2 = local hyperaemia, inflammation, and oedema; 3 = hyperaemia, extensive oedema, and local haemorrhage; 4 = extensive haemorrhage; 5 = haemorrhage, local necrosis and pneumatosis intestinalis; and 6 = extensive necrosis, haemorrhage and pneumatosis intestinalis, Fig. 1c).^25^ A score of at least 3 in any of the four regions, resulted in a pig diagnosed with NEC. This cut-off for NEC diagnosis included pigs with only local haemorrhagic lesions (e.g. initial, mild, NEC-like lesions).^25^ Colonic contents were collected from loops across the colon and stored at −80 °C for later microbiota analysis.

A series of intestinal parameters were recorded during experiments. Body weight was recorded daily. On 1 day before euthanization, intestinal hexose absorptive capacity was evaluated by galactose concentration in plasma. Before euthanization, intestinal permeability evaluated by lactulose/mannitol ratio (Lac/Man) in urine was recorded. At euthanization, mucosal villus height/crypt depth ratio (villus/crypt) and activities of brush border enzymes, including lactase, maltase, sucrase, aminopeptidase N (ApN), aminopeptidase A (ApA), and dipeptidyl peptidase IV (DPPIV) in proximal (Prox), middle (Mid) and distal (Dist) regions of small intestine and intestinal mucosal gene expression of Toll-like receptor 4 (TLR4, a bacterial receptor often associated with NEC), was measured and reported as previously described.^23,24^ In the present study, only data from pigs with colonic content available for GM analysis (53 of 74 piglets in MS study; all pigs, *n* = 57 in MM study) are reanalysed and used for correlation analyses to GM effects.

### 16S ribosomal RNA gene amplicon sequencing

DNA extraction of about 200 mg colonic content from each pig was processed by Bead-Beat Micro AX Gravity Kit (A&A, Gdynia, Poland) according to the manufacturer’s instructions. Library preparation was conducted according to a published protocol.^26^ The V3 hypervariable region was chosen for a two-step PCR amplicon using primers NXT_388_F: 5′-CCTACGGGWGGCAGCAG-3′ and NXT_518_R: 5′-ATTACCGCGGCTGCTGG-3′ (Integrated DNA Technologies, Leuven, Belgium) and followed by 150 bp pair-end NextSeq sequencing (Illumina, CA). Following our in-house procedures of bioinformatics processing,^27^ raw sequencing reads were merged and trimmed, and chimera was removed to generate zero-radius Operational Taxonomic Units (zOTUs) using the UNOISE3^28^ algorithm implemented in Vsearch (version 2.21.1).^29^ The Greengenes database (13.8)^30^ served as taxonomic annotation reference. In the MS study, the mean and median sequencing depth of all samples was 70,968 and 70,865 reads, and 1513 zOTUs were identified across all samples. In the MM study, the mean and median sequencing depth of all samples were 66,382 and 70,721 reads, and 2020 zOTUs were detected across all samples.

### Statistics

Binary outcomes were presented as counts (percentages) and analysed by logistic regression models and continuous variables were presented as means (and standard deviation) and analysed by linear regression models. For post hoc tests, Tukey’s test was used in the MS study and Dunnett’s test was used in the MM study with the C0 group as reference. Litter was included as a fixed effect factor and adjusted for in all statistical analyses. Pre-analyses showed that sex and birth weight did not affect the GM and these variables were subsequently excluded from the statistical analyses.

The GM data was analysed using the R packages Phyloseq^31^ and Vegan.^32^ The zOTUs were rarefied at 3000 counts per sample, with all samples preserved. Species diversity (alpha-diversity) was estimated based on the Shannon index and the number of observed zOTUs, and comparisons between groups were conducted by linear regression. Post hoc tests were conducted as above. The compositional similarity of the microbial community (beta-diversity) was assessed based on the weighted UniFrac and unweighted UniFrac distance metrics and statistical difference between groups were determined by permutational multivariate analysis of variance (PERMANOVA). Genera with a mean of relative abundance over 1% and presence in over 10% of samples were selected for testing the difference in relative abundance between treatment groups by DESeq2.^33^ Correlations between the relative abundance of genera (the top ten most abundant genera in each group) and daily weight gain, as well as the intestinal health parameters were conducted by Spearman’s correlation across feeding groups within each experiment. Statistical differences of species diversity, microbial community structure and differential genera between pigs with and without NEC were determined by linear regression, PERMANOVA and DESeq2, respectively, with adjustment for litter and feeding as fixed effect factors. False discovery rate (FDR) correction for multiple testing was applied. Effects with *p* < 0.05 were regarded as statistically significant. A correlation coefficient larger than 0.50 with missing data in less than 30% of all samples was deemed to be correlated. All statistical analyses and visualisations were performed by R (version 4.2.1).

## Results

### Intestinal lesions, structure and function

In the MS study, pigs were fed formula or colostrum until day 5, followed by euthanasia, or continued with the same feeding or shifted to another feeding until day 9. The diet groups did not induce significant differences in overall NEC diagnosis, neither on day 5 or 9, with most NEC pigs diagnosed with mild NEC scores (all *p* > 0.05, Supplementary Table 2). However, there were marked differences in several markers of intestinal structure and function, most pronounced in the proximal and middle regions of small intestine. On day 9, intestinal structure, indicated by villus/crypt ratio, was higher in the proximal region of small intestine in the groups given BC from birth, compared with the group given exclusive formula (CC, CF vs. FF, both *p* < 0.05, Supplementary Table 3). In terms of intestinal function, no significant difference in absorptive capacity, as measured by plasma galactose concentration, was found between different groups on day 9 (all *p* > 0.05, Supplementary Table 3), while intestinal permeability, as indicated by urinary lactulose/mannitol ratio, was lower in the groups that received BC, compared with the group fed exclusive formula (CC, CF, FC vs. FF, all *p* < 0.05, Supplementary Table 3). Similarly, activities of lactase, ApN, ApA and DPPIV were significantly increased by colostrum supplementation (CC, CF, FC vs. FF, all *p* < 0.05, Supplementary Table 3). The activity of sucrase was only significantly higher in the pigs receiving exclusive colostrum, compared with pigs fed exclusive formula (*p* < 0.05, Supplementary Table 3). No significant differences in TLR4 gene expressions were found (all *p* > 0.05, Supplementary Table 3).

In the MM study, pigs were fed different proportions of colostrum with formula within the same day until day 5. The highest incidence of NEC was found in the pigs exclusively fed formula (C0) but overall, there was no significant difference in NEC incidence among groups (most NEC scores 3-4, e.g. mild, *p* > 0.05, Supplementary Table 2). Villus/crypt ratio was higher in the C75 and C50 groups in the proximal region of small intestine (C75, C50 vs. C0, all *p* < 0.05, Supplementary Table 4), compared with the C0 group (*p* < 0.05). All details of the intestinal structure and function effects were reported previously,^24^ with higher plasma galactose concentration, higher activities of sucrase, lactase, ApA, ApN and DPPIV, and lower urinary lactulose/mannitol ratios observed in the C75 and C50 pigs compared with the C0 ones (all *p* < 0.05, Supplementary Table 4). Likewise, gene expression of TLR4 was downregulated in the C75 group, compared with the C0 group (*p* < 0.05, Supplementary Table 4).

### GM when feeding colostrum alone, before or after formula (MS study)

**Fig. 2:**
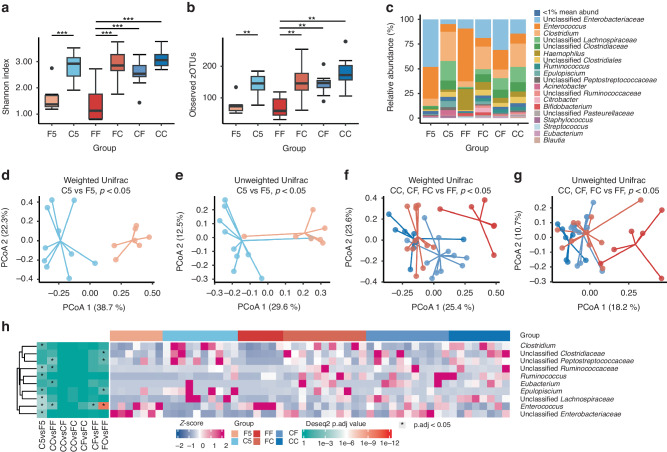
Feeding bovine colostrum (C) before or after formula (F) altered the gut microbiota in preterm pigs. **a**, **b** Feeding some colostrum (CC, CF and FC) increased the bacterial species diversity. **c** Across groups, the microbial composition was dominated by unclassified *Enterobacteriaceae, Enterococcus* and *Clostridium*. **d**–**g** Pigs fed exclusively formula (F5, FF) generally had a different microbial community structure than the groups fed partial or exclusive bovine colostrum, which were similar (C5, CF, FC, CC). **h** Colostrum feeding reduced *Enterococcus* density on day 5 (C5 vs. F5) and prevented a rise in *Enterococcus* density in pigs fed formula after day 5 (CF vs. FF). The heatmap illustrates the differential microbiota genera among groups. Differentially abundant core species (mean relative abundance > 1% among at least 10% of samples) are shown. *, *p* < 0.05; **, p < 0.01; ***, *p* < 0.001.

On day 5, species diversity as assessed by the Shannon index and the number of observed zOTUs, was higher in the C5 group, compared with the F5 (both *p* < 0.05, Figs. 2a, b). Microbial community structure differed between the C5 and F5 groups as well as determined by both weighted UniFrac and unweighted UniFrac distances (both *p* < 0.05, Figs. 2d, e).

On day 9, species diversity was higher in the groups receiving any colostrum, compared with the FF group fed exclusive formula (FC, CF, CC vs. FF, all *p* < 0.05, Figs. 2a, b). Groups fed exclusively colostrum or formula showed different microbial community structure (CC vs. FF, both *p* < 0.05, Figs. 2f, g). Feeding colostrum after formula resulted in a microbial community structure that resembled the one after exclusive colostrum feeding (FC vs. CC, both *p* > 0.05; FC vs. FF, both *p* < 0.05, Figs. 2f, g). Feeding formula after colostrum did not lead to a community structure similar to that observed in the pigs on formula only (CF vs. CC, weighted UniFrac *p* < 0.05, unweighted UniFrac *p* > 0.05; CF vs. FF, both *p* < 0.05, Figs. 2f, g). FC and CF pigs only had significant difference in microbial community structure by the weighted UniFrac distances (*p* < 0.05, Fig. 2f), but not by the unweighted UniFrac one (*p* > 0.05, Fig. 2g).

*Enterococcus* and *Clostridium* and an unclassified group of *Enterobacteriaceae* were the most abundant genera across groups on days 5–9 (Fig. 2c). The relative abundance of *Enterococcus* was higher in the exclusive formula groups, compared with the groups that had any colostrum (F5 vs. C5; FF vs. CC, FC, CF, all *p* < 0.05, Fig. 2h, Supplementary Fig. 1a). Higher relative abundance of *Clostridium*, *Ruminococcus*, *Epulopiscium* and unclassified *Lachnospiraceae*, and a lower relative abundance of unclassified *Enterobacteriaceae* were found in the pigs exclusively fed colostrum until day 5 (C5 vs. F5, both *p* < 0.05, Fig. 2h, Supplementary Fig. 1b–d), but not on day 9 (CC vs. FF, both *p* > 0.05, Fig. 2h). The relative abundance of *Eubacterium* and unclassified *Peptostreptococcaceae* was higher in CC versus FF pigs on day 9 (all *p* < 0.05, Fig. 2h). The relative abundance of unclassified *Clostridiaceae* was higher in FC versus FF pigs (*p* < 0.05, Fig. 2h, Supplementary Fig. 1e). More genera were altered when shifting to colostrum feeding (FC vs. FF, four genera) than the opposite direction (CF vs. FF, one genus, Fig. 2h).

**Fig. 3:**
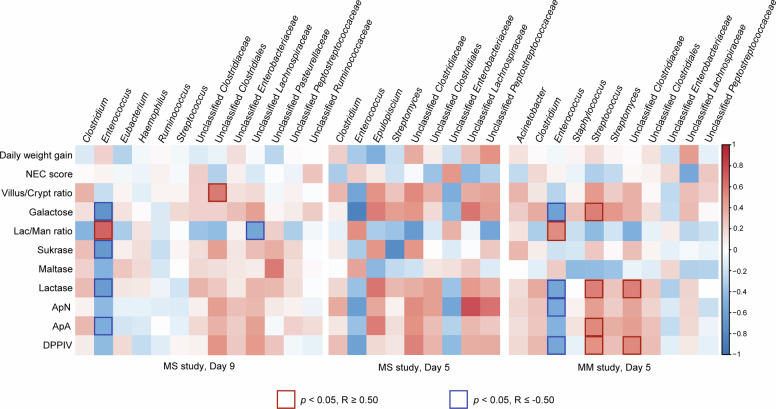
Correlation analyses between microbiota composition and intestinal parameters. Probability (*p*) values <0.05 for correlation between gut microbiota and parameters of daily weight gain, intestinal structure (villous/crypt ratio in proximal intestine, Lac/Man ratio) and function (galactose levels, enzyme activities in the proximal intestine). MS study, day 9 (left): After FDR correction, *Enterococcus* was negatively correlated with galactose absorptive capacity and sucrase, lactase and ApA activities, but positively correlated with permeability (Lac/Man ratio). MS study, day 5 (middle): No significant correlation was found between gut microbiota and parameters of intestinal structure and function. MM study, day 5 (right): After FDR correction, intestinal galactose absorptive capacity and enzyme activities (lactase, peptidases) were negatively correlated with *Enterococcus* but positively correlated with *Streptococcus* and unclassified *Clostridiaceae*. Colour red represents positive correlations and blue represents negative correlations. Most dense colour shows highest correlation coefficient; squares show *p* values remaining significant after FDR correction (R ≤ −0.5, R ≥ 0.5).

**Fig. 4:**
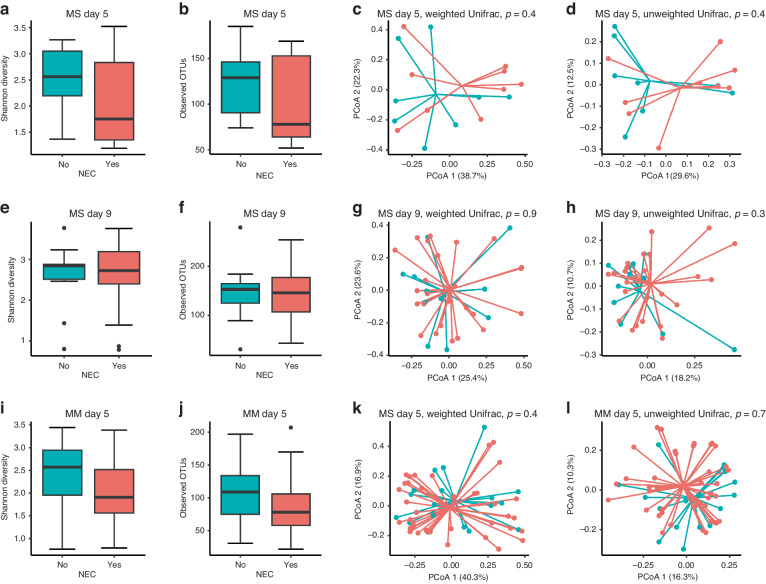
Association analyses between the gut microbiota and NEC diagnosis. No significant differences in species diversity or microbial community structure were found between groups of pigs with or without NEC in the MS study on day 5 (**a**–**d**) or day 9 (**e**–**h**), or in the MM study on day 5 (**i**–**l**).

Correlation analyses between the top ten abundant GM members and specific intestinal variables (Fig. 3) showed that the relative abundance of *Enterococcus* was negatively correlated with plasma galactose, and proximal lactase, sucrase and ApA, middle lactase and ApA activities, while positively correlated with the lac/man ratio on day 9 (all *p* < 0.05, Fig. 3). Correlations with variables recorded in the middle and distal intestine were less pronounced than in the proximal intestine (data not shown). No statistical correlation was found on day 5 in the MS study after FDR correction (all *p* > 0.05), however again *Enterococcus* abundance tended to correlate negatively with galactose absorption (*p* = 0.06, Fig. 3). No significant correlations were observed between NEC severity score and predominant GM members, including *Enterococcus* (*p* > 0.05, Fig. 3). No significant differences in species diversity, community structure or specific genus abundance were found between pigs with or without NEC diagnosis across feeding groups (all *p* > 0.05, Figs. 4a–h) on day 5 (*n* = 17) and 9 (*n* = 36).

### GM after feeding variable proportions of colostrum in the same day (MM study)

**Fig. 5:**
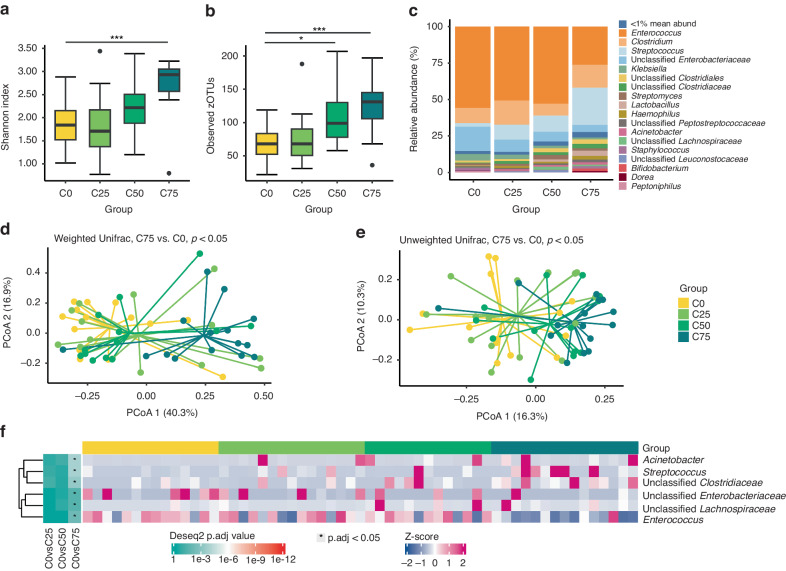
Minimum 50% of the daily meals being bovine colostrum (C) changes the gut microbiota. **a**, **b** More than 50% colostrum per day increased species diversity. **c** Across groups, the microbial composition was dominated by *Enterococcus, Clostridium* and *Streptococcus*. **d**, **e** Feeding more than 50% colostrum significantly affected the microbiome community structure. **f** 75% of daily meals being colostrum decreased the relative abundance of *Enterococcus* and unclassified *Enterobateriaceae* relative to exclusive formula feeding. The heatmap illustrates the differential microbiota genera among groups. Differentially abundant core species (mean relative abundance > 1% among at least 10% of samples) are shown. *, *p* < 0.05; **, p < 0.01; ***, *p* < 0.001.

Species diversity was higher in C75 than C0 pigs, as indicated both by the Shannon index and the number of zOTUs (both *p* < 0.01, Figs. 5a, b). Only the number of observed OTUs was higher in C50 versus C0 pigs (*p* < 0.05, Fig. 5b). A significant difference in microbial community structure was found between the C75 and C0 groups (both *p* < 0.05, Figs. 5d, e) and between the C50 and C0 groups (unweighted UniFrac distance, *p* < 0.05, Fig. 5e).

*Enterococcus*, *Clostridium*, *Streptococcus* and a group of unclassified *Enterobacteriaceae* were the four most abundant genera across groups (Fig. 5c). Relative abundance of *Enterococcus* and unclassified *Enterobacteriaceae* was lower, while *Acinetobacter*, *Streptococcus*, unclassified *Clostridiaceae* and unclassified *Lachnospiraceae* higher in C75 relative to C0 pigs (all *p* < 0.05, Fig. 5f, Supplementary Fig. 1f–j). No significant difference in the relative abundance of any genus was found between C50 or C25 versus C0 (all *p* > 0.05, Fig. 5f).

When correlating specific GM bacterial species and intestinal variables, most significant correlations were again found in variables in the proximal intestine (data not shown for middle and distal intestine). A negative correlation was observed between the relative abundance of *Enterococcus* and plasma galactose, proximal lactase, ApN and DPPIV activities (all *p* < 0.05, Fig. 3). Conversely, a positive correlation was found between the relative abundance of *Streptococcus* and galactose levels, proximal ApA and DPPIV activities (all *p* < 0.05, Fig. 3) and middle lactase activities (data not shown), as well as between unclassified *Clostridiaceae* and proximal lactase and DPPIV activities (all *p* < 0.05, Fig. 3). No significant correlations were found between the NEC severity score and the abundance of the top ten most abundant genera, including *Enterococcus* (*p* > 0.05, Fig. 3). Like in the MS study, no significant differences in species diversity, microbial community structure or abundance of specific genus were found between pigs with and without NEC diagnosis in the MM study (all *p* > 0.05, Figs. 4i–l).

## Discussion

The present study is a follow-up study on colonic GM changes in formula-fed preterm pigs derived from two previous studies demonstrating clear benefits of supplemental bovine colostrum on intestinal health parameters.^23,24^ Here, we documented marked effects on the GM of feeding bovine colostrum alone, before or after formula feeding. We demonstrated limited statistical correlation between the diet-related GM effects and the initial NEC-like lesions or gut maturation markers in preterm pigs. It appears that intestinal factors were more important than the GM composition to determine gut NEC resistance in the first weeks of life. While the exact phenotype(s) of NEC and its age- and diet-related development differ between preterm pigs and infants, the overall effect of diet and the GM on NEC sensitivity could be similar. We conclude that the bovine colostrum-related gut protection in the first week of life of preterm neonates does not primarily occur via benefits to GM composition.

A large number of previous studies in preterm pigs have documented marked NEC-preventive and gut maturational effects of feeding exclusive bovine colostrum, relative to infant formula.^6,8,10,11,34^ However, the effects on the GM were not entirely consistent across these studies and for both infants and pigs, it remains unclear if NEC-related GM changes are the cause or consequence of NEC progression.^3,17,18^ It is important to know if bovine colostrum retains any benefits together with formula feeding, as diet shifts or supplementing formula meals with colostrum are likely clinical scenarios when attempting to use bovine colostrum in preterm infants.^12–14,35^ The proposed beneficial effects of bovine colostrum on gut maturation and the GM are related to the high level of antimicrobial and immunomodulatory compounds.^5^ We previously showed that partial colostrum feeding (before/after/during formula feeding period) improved a range of intestinal parameters, although with limited effects on early NEC-like lesions.^23,24^ Now we show that the colonic microbiota in such pigs is similar to that of pigs fed exclusively colostrum for 9 days and differs from pigs fed exclusive formula (MS study). If both bovine colostrum and formula meals are provided on the same day (MM study), at least half of the meals should be colostrum to observe GM effects (e.g. preventing overgrowth of *Enterococcus*). Strikingly, such diet-induced GM compositional changes were not clearly associated with early NEC-like lesions, but there were significant correlations with certain markers of intestinal function. This finding suggests that the GM participates in regulating intestinal maturation in early life but might not critically determine early onset of NEC, at least not in these preterm pigs.

The results suggest that bovine colostrum has potential to alter the GM and intestinal parameters, both when fed before or after a few days of formula feeding (MS study). The benefits of bovine colostrum is obvious during the first week after preterm birth but could last longer, in line with our previous studies.^8,36^ Possibly, bovine colostrum can be fed before/after formula to prevent/repair formula-induced GM disturbance and intestinal dysfunction. While the microbial community structure of the FC group was similar to that of the CC group, specific differences were observed between the CF and CC pigs. A similar pattern was noted in the bacterial genera, with the majority of differences found between the FC and FF groups, rather than between the CF and FF groups. This implies that the impact of colostrum on the GM was more pronounced when provided after formula feeding (FC group), somewhat in contrast to our previous observations on systemic immunity and gut health in the CF pigs.^23^

Abundance of several genera within the GM changed in response to colostrum feeding. In preterm pigs, the dominant bacterial genera identified in the GM were also found in preterm infants during early life, including *Enterococcus*,^37^ which is less abundant in term infants.^38^ The most remarkable effect of bovine colostrum feeding on specific bacterial genera was the inhibition of *Enterococcus*. *Enterococcus* was the most abundant genus in the exclusively formula-fed groups (F5 and FF), relative to all other groups (C5, CC, CF and FC) and bovine colostrum effectively reduced abundance of *Enterococcus*, regardless of formula feeding. Our pilot trials in preterm infants also showed bovine colostrum reduced abundance of *Enterococcaceae*, relative to DHM,^35^ and in mice, it inhibited proliferation of *Escherichia coli* and *Enterococcus* spp.^39^ *Enterococcus* contains facultative pathogens which have been associated with systemic infections and NEC in infants via positive blood cultures.^17^ However, whether diet-induced inhibition of gut *Enterococcus* protects preterm infants from NEC and intestinal dysfunction is not known.

When alternating colostrum and formula meals within the same day (MM study), bovine colostrum feeding increased the species diversity and affected GM community structure, but only when more than half of meals were colostrum. These changes were mainly driven by the high-abundant taxa present in the group fed 75% colostrum, suggesting that colostrum effects on the GM were highly dose-dependent. When bovine colostrum was a lower proportion of daily meals, its impacts on the GM effects was limited. Correspondingly, the highest intestinal nutrient absorption, enzyme activity levels and villus heights, and lowest permeability, crypt depths, cytokine levels and TLR4 expressions were found in the C75 pigs, followed by the C50 pigs, as reported in our earlier studies.^24^ Dose-response relationships were present for colostrum effects on epithelial inflammation in an in vitro study.^40^ The reduction in *Enterococcus* and *Enterobacteriaceae* (a family of Gammaproteobacteria) in the pigs fed 75% colostrum (improved intestinal health) is in accordance with their high abundance in infant NEC,^41^ yet cause-effect relationships are unclear. Also, for the MM study, we observed no association between *Enterococcus* abundance and NEC diagnosis, but tendencies to negative correlations with parameters of intestinal maturation. It cannot be excluded that increased density of *Streptococcus* and unclassified *Clostridiaceae* in the pigs fed 75% colostrum positively affected the immature intestine^3,42,43^ but this remains speculative, and the exact species within these genera or families were not determined. Importantly, feeding 75% of daily meals as bovine colostrum in the first week of life is clinically very challenging due to concerns of protein overload, as shown in our clinical trials.^12,13,44^ However, our results from preterm pigs are consistent with the observations in infants that bovine colostrum exerts clinical benefits to feeding intolerance (a surrogate marker of gut maturation) only if volumes exceed those of formula in the first 10 days of life.^13^

Several infant studies reported reduced GM species diversity related to NEC^45^ and increased abundance of Gammaproteobacteria.^41,46^ Most studies investigate infant GM during severe NEC outbreaks and under the influence of antibiotics and enteral feeding withdrawal. Thus, such studies cannot easily be used to define whether early-life GM changes predispose to later NEC. In preterm pigs, we detected no association between GM (diversity, relative abundance of specific bacterial genera) and NEC diagnosis, consistent with our earlier pigs studies and also with some trials in infants.^6,8,47–49^ Instead, the negative correlations between *Enterococcus* and maturation parameters of the proximal small intestine might suggest that *Enterococcus* overgrowth could be used as a marker of formula-induced intestinal dysfunction and early NEC lesions, although direct causal links are questionable. Elevated *Enterococcus* abundance paralleled elevated intestinal permeability in preterm pigs receiving predominantly formula. *Enterococcus faecalis* is known to impair epithelial barrier function by gelatinase, increasing intestinal permeability and inflammation.^50^ In turn, such inflammation may affect brush border enzyme activities and nutrient uptake capacity. While diet-induced changes to GM may influence intestinal pathways eventually leading to NEC, such GM effects represent only one of many factors, that together with intestinal host factors determine whether NEC develops.

A prominent bioactive component of BC is Igs, particularly IgG.^5^ Colostral bovine IgG may act locally in the gut lumen of both pigs and infants by binding specific pathogens and modulating mucosal immune responses.^51–53^ Lactoferrin, another key component of bovine colostrum, has been shown to increase species diversity and change microbial community structure in infants when combined with milk fat globule membrane (MFGM) in infant formula,^54^ and promote intestinal cell maturation in vitro.^55,56^ Its iron-binding ability can impede pathogen access to essential iron^57^ and stimulate growth of bacteria requiring less iron, such as lactobacilli and *Bifidobacterium*^58^ that may play pivotal roles in the early phase of gut colonisation.^37^ Stool consistency and intestinal motility affects gut colonisation in both infants and adults^58–60^ and supplementation of bovine colostrum to human milk of preterm infants tended to increase stool frequency in a recent study.^60^ Therefore, the GM alterations in our studies could be attributed in part to diet-related effects on food passage, in addition to the specific effects of milk nutrients and bioactives in bovine colostrum versus infant formula.

Our study has strengths but also several limitations. Preterm, caesarean-delivered pigs allow detailed experimental control and strict feeding regimens to assess influences of interacting host and environmental factors on the GM, NEC sensitivity and intestinal functions. While differences in NEC phenotypes and GM colonisation clearly exist between preterm pigs and infants, the factors that predispose to NEC (prematurity, formula feeding, gut colonisation) are highly similar.^25,61^ Pig studies allow insights into early-developing NEC lesions and the possible contributing factors to NEC, not only its consequences. Yet, it remains difficult to directly compare preterm pig and infant GM development due to numerous differences in gestational age, postnatal age, diets, environment and sampling methods across preterm pig and infant studies. Our feeding regimens demonstrated proof-of-principle for combinations of bovine colostrum and infant formula, but many more combinations with regards to the timing, amounts and preparation of diets (e.g. mixing different diets into the same meal) are relevant for preterm infants. Further, we did not investigate bovine colostrum in combination with relevant milk diets, such as DHM and MOM that are frequently supplied in clinical practices for preterm infants and may affect the GM and intestinal development differently than formula. Our conclusions regarding clinical and physiological effects of specific bacterial strains and species are restricted by the relatively crude GM characterisation, low sample size, and lacking samples from intestinal lumen and mucosa. However, we conclude that feeding bovine colostrum before, during or after formula feeding affects GM colonisation in preterm piglets (e.g. higher microbial diversity, less *Enterococcus*), but that diet-related GM effects are unlikely the key factor for NEC induction or prevention in the first 1–2 weeks of life.

## Supplementary information

Supplementary information

## Data Availability

The datasets generated during the current study are available from the corresponding author upon reasonable request.
