# Supplementary material for: Bovine colostrum prevents formula-induced gut microbiota dysbiosis in preterm pigs
Source: Pediatr Res. 2024 Jul 8;97(2):818–26. doi: 10.1038/s41390-024-03379-x (PMC12014488; doi:10.1038/s41390-024-03379-x)
Supplement: Supplementary file 1 — Supplementary information [file 41390_2024_3379_MOESM1_ESM.pdf]

**Supplementary Table 1.** The composition of bovine colostrum and formula used in the experiments

| Constituents, per liter | MS | | MM | |
|---|---|---|---|---|
| | Bovine colostrum | Formula | Bovine colostrum | Formula |
| Phlexy-Vits, g | - | - | - | 2 |
| SHS Seravit, g | - | 12 | - | - |
| SHS Liquigen MCT, ml | - | 43 | - | 41 |
| Nutricia Calogen LCT, ml | - | 30 | - | 29 |
| Nutricia Fantomalt, g | - | 18 | - | 33 |
| WPC (DI-9224), g | - | 70 | - | 67 |
| Casein (Miprodan 40), g | - | 35 | - | 33 |
| ColoDan powder, g | 170 | - | 170 | - |
| Nutritional value | | | | |
| Energy, kJ | 3405 | 3398 | 3351 | 3356 |
| Protein, g | 92.2 | 92.4 | 88 | 88 |
| Whey, g | 62 | 62 | 59 | 59 |
| Casein, g | 30 | 30 | 29 | 29 |
| Fat, g | 37.4 | 37.1 | 35.5 | 35.5 |
| Carbohydrate, g | 26.7 | 26.5 | 31.6 | 31.9 |
| Maltodextrin, g | - | 24 | - | 29.9 |
| Lactose, g | 26.7 | - | 31.6 | 2 |
| Other, g | - | 2 | - | - |

**Supplementary Table 2.** NEC score in the MS and MM studies

| Study | Group | NEC score | | | | | | NEC (%) (score 3-6) |
|---|---|---|---|---|---|---|---|---|
| | | 1 | 2 | 3 | 4 | 5 | 6 | |
| MS day 5 | | | | | | | | |
| | C5 (n=10) | 6 | 0 | 2 | 2 | 0 | 0 | 4 (40) |
| | F5 (n=7) | 0 | 2 | 1 | 4 | 0 | 0 | 5 (71) |
| MS day 9 | | | | | | | | |
| | CC (n=8) | 3 | 1 | 4 | 0 | 0 | 0 | 4 (50) |
| | CF (n=11) | 0 | 2 | 7 | 2 | 0 | 0 | 9 (82) |
| | FC (n=11) | 3 | 1 | 6 | 1 | 0 | 0 | 7 (64) |
| | FF (n=6) | 0 | 1 | 5 | 0 | 0 | 0 | 5 (83) |
| MM day 5 | | | | | | | | |
| | BC0 (n=14) | 1 | 2 | 3 | 5 | 2 | 1 | 11 (79) |
| | BC25 (n=15) | 3 | 1 | 2 | 4 | 1 | 4 | 11 (73) |
| | BC50 (n=13) | 3 | 2 | 1 | 7 | 0 | 0 | 8 (62) |
| | BC75 (n=15) | 5 | 1 | 6 | 2 | 1 | 0 | 9 (60) |

**Supplementary Table 3.** Parameters of intestinal structure and function in the MS study (means ± SD)

| | Day 5 | | *p* value C5-F5 | Day 9 | | | | *p* value | | | | | |
|---|---|---|---|---|---|---|---|---|---|---|---|---|---|
| | C5 (n=10) | F5 (n=7) | | CC (n=8) | CF (n=11) | FC (n=11) | FF (n=6) | CC-CF | CC-FC | CC-FF | CF-FC | CF-FF | FC-FF |
| Daily weight gain (g/kg) | 22.76 （8.74） | 10.64 （11.41） | * | 30.80 (16.42) | 32.22 (12.86) | 22.47 (12.66) | 33.53 (11.33) | ns | ns | ns | ns | ns | ns |
| Villus/crypt ratio[#] | 6.3 (1.2) | 4.1 (1.4) | ** | 5.9 (1.9) | 5.2 (1.1) | 4.51 (1.2) | 3.1 (1.4) | ns | ns | * | ns | * | ns |
| Galactose, g/L | 1244 (752) | 363 (486) | ns | 868 (726) | 892 (555) | 560 (549) | 93 (100) | ns | ns | ns | ns | ns | ns |
| Lactulose/ Mannitol ratio | 0.04 (0.06) | 0.09 (0.05) | ns | 0.01 (0.01) | 0.06 (0.03) | 0.06 (0.07) | 0.15 (0.08) | ns | ns | ** | ns | * | * |
| Sucrase (U/g) | 0.27 (0.12) | 0.21 (0.12) | ns | 1.12 (0.87) | 0.56 (0.25) | 0.77 (0.44) | 0.22 (0.10) | ns | ns | * | ns | ns | ns |
| Maltase (U/g) | 2.1 (1.0) | 2.7 (1.8) | ns | 5.84 (3.42) | 6.16 (4.80) | 8.57 (5.85) | 2.43 (1.61) | ns | ns | ns | ns | ns | * |
| Lactase (U/g) | 35.7 (13.4) | 13.2 (16.5) | * | 32.2 (17.3) | 20.8 (12.7) | 22.9 (13.7) | 5.0 (5.6) | ns | ns | *** | ns | ** | ** |
| Aminopeptidase N (U/g) | 6.32 (2.12) | 2.70 (1.68) | ** | 7.44 (2.59) | 7.74 (3.81) | 6.06 (2.47) | 2.69 (1.53) | ns | ns | * | ns | * | ns |
| Aminopeptidase A (U/g) | 2.06 (0.60) | 1.17 (1.10) | ns | 2.31 (0.77) | 1.89 (0.67) | 1.61 (0.53) | 0.72 (0.41) | ns | * | *** | ns | ** | * |
| Dipeptidylpeptidase IV (U/g) | 2.10 (0.96) | 2.70 (1.77) | ** | 2.61 (0.71) | 2.62 (0.94) | 2.25 (0.84) | 0.91 (0.56) | ns | ns | ** | ns | ** | ** |
| TLR4/C5 mRNA expression ratio | 1 | 2.25 (2.75) | ns | 4.91 (5.26) | 3.99 (3.31) | 5.94 (5.00) | 5.39 (4.19) | ns | ns | ns | ns | ns | ns |

[#] For villous structure, enzyme activities and gene expression, only values from proximal region of small intestine are presented.

ns $p \geq 0.05$, * $p < 0.05$, ** $p < 0.01$, *** $p < 0.001$.

**Supplementary Table 4.** Parameters of intestinal structure and function in the MM study (means ± SD)

| | C0 (n=14) | C25 (n=15) | C 50 (n=13) | C75 (n=15) | *p* value | | |
|---|---|---|---|---|---|---|---|
| | | | | | C25-C0 | C50-C0 | C75-C0 |
| Daily weight gain (g/kg) | 11.28 (10.84) | 9.57 (16.24) | 24.08 (6.72) | 23.52 (16.52) | ns | * | * |
| Villus/crypt ratio[#] | 4.27 (1.16) | 5.04 (1.88) | 6.48 (2.54) | 7.43 (1.55) | ns | ** | *** |
| Galactose (g/L) | 301.6 (345.6) | 634.9 (707.8) | 804.1 (510.9) | 1357.3 (631.8) | ns | * | *** |
| Lactulose/Mannitol ratio | 0.19 (0.15) | 0.08 (0.03) | 0.05 (0.05) | 0.03 (0.03) | ns | ** | *** |
| Sucrase (U/g) | 0.22 (0.09) | 0.25 (0.09) | 0.24 (0.06) | 0.32 (0.11) | ns | ns | * |
| Maltase (U/g) | 2.85 (1.76) | 2.37 (1.12) | 2.20 (0.65) | 1.83 (0.77) | ns | ns | ns |
| Lactase (U/g) | 6.32 (6.77) | 23.65 (19.95) | 18.12 (16.50) | 40.28 (16.64) | ** | * | *** |
| Aminopeptidase N (U/g) | 1.96 (0.96) | 3.88 (2.60) | 3.28 (1.64) | 4.30 (1.23) | ** | ns | *** |
| Aminopeptidase A (U/g) | 0.66 (0.51) | 1.83 (1.34) | 1.72 (1.36) | 2.04 (0.54) | ** | * | *** |
| Dipeptidylpeptidase IV (U/g) | 0.95 (0.43) | 1.46 (0.61) | 1.44 (0.56) | 1.69 (0.28) | * | * | *** |
| TLR4/C0 mRNA expression ratio | 1 (0.33) | 0.89 (0.61) | 0.89 (0.33) | 0.61 (0.28) | ns | ns | * |

Results are cited from previous publication[26].

[#] For villous structure, enzyme activities and gene expression, only values from proximal region of small intestine are presented.

ns $p \geq 0.05$, * $p < 0.05$, ** $p < 0.01$, *** $p < 0.001$.

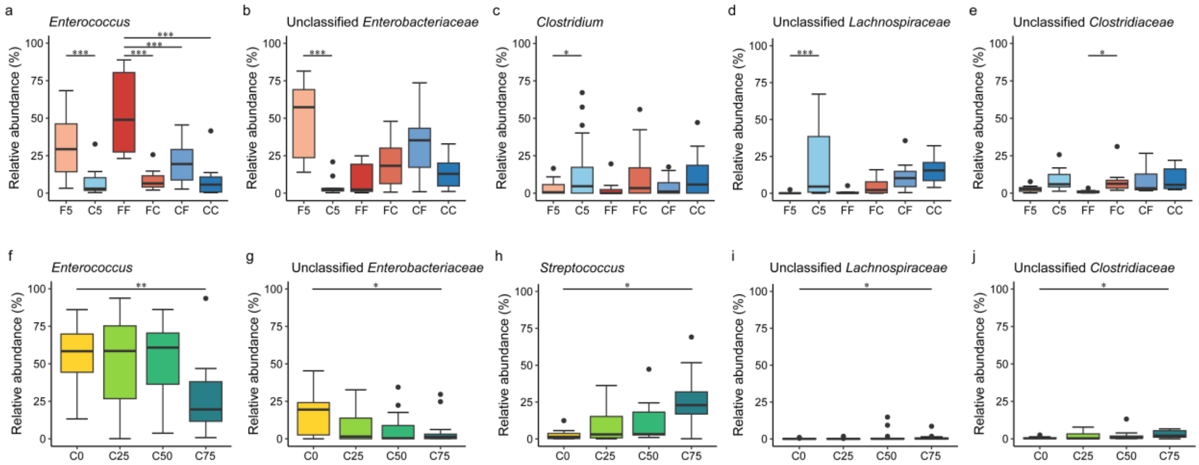


**Supplementary Figure 1**. The five most abundant genera among the differential genera between groups in the MS study (a-e) and the MM study (f-j). *Enterococcus* was consistently reduced in groups receiving colostrum (a) and most significantly reduced in the C75 group (f).
